# Dietary cystine level affects metabolic rate and glycaemic control in adult mice^☆^^☆☆^

**DOI:** 10.1016/j.jnutbio.2010.12.009

**Published:** 2012-04

**Authors:** Amany K. Elshorbagy, Chris Church, Maria Valdivia-Garcia, A. David Smith, Helga Refsum, Roger Cox

**Affiliations:** a Department of Pharmacology, University of Oxford, UK; b Department of Physiology, Faculty of Medicine, University of Alexandria, Egypt; c MRC Harwell, Metabolism and Inflammation, Harwell Science and Innovation Campus, Harwell, UK; d Department of Nutrition, Institute of Basic Medical Sciences, University of Oslo, Norway

**Keywords:** Cysteine, Cystine, Sulfur amino acids, Adiposity, PTP1b, Metabolic rate, Energy expenditure, Glucose tolerance, Insulin sensitivity

## Abstract

Plasma total cysteine (tCys) is strongly and independently associated with obesity in large human cohorts, but whether the association is causal is unknown. Dietary cyst(e)ine increases weight gain in some rodent models. We investigated the body composition, metabolic rate and metabolic phenotype of mature C3H/HeH mice assigned to low-cystine (LC) or high-cystine (HC) diets for 12 weeks.

Compared to LC mice, HC mice gained more weight (*P*=.004 for 12-week weight gain %), with increased fat mass and lean mass, and lowered O_2_ consumption and CO_2_ production by calorimetry. The HC mice had 30% increase in intestinal fat/body weight % (*P*=.003) and ∼twofold elevated hepatic triglycerides (*P*=.046), with increased expression of hepatic lipogenic factors, peroxisome proliferator-activated receptor-γ and sterol regulatory element binding protein-1. Gene expression of both basal and catecholamine-stimulated lipolytic enzymes, adipose triglyceride lipase and hormone-sensitive lipase was inhibited in HC mice adipose tissue. The HC mice also had elevated fasting glucose (7.0 vs. 4.5 mmol/L, *P*<.001) and a greater area under the curve (*P*<.001) in intraperitoneal glucose tolerance tests, with enhanced expression of the negative regulator of insulin signaling, protein tyrosine phosphatase-1B, in liver and adipose tissue.

Overall, high cystine intake promotes adiposity and an adverse metabolic phenotype in mice, indicating that the positive association of plasma tCys with obesity in humans may be causal.

## 1 Introduction

Cysteine is a proteinogenic sulfur amino acid that is associated with cardiovascular disease [1,2] and metabolic syndrome [3] in humans. Cysteine is obtained from diet and synthesized from the methionine product, homocysteine, by transsulfuration, with cystathionine as an intermediate [4]. In addition to protein synthesis, cysteine is utilized in synthesis of taurine and coenzyme A, and the antioxidant glutathione (Supplementary Figure 1) [4]. Due to its sulfhydryl group reactivity, cysteine can exist in reduced (cysteine) or disulfide (cystine) forms, or as a mixed disulfide with other sulfhydryl compounds.

Plasma total cysteine (tCys) concentrations consistently correlate with body mass index (BMI), fat mass and odds of obesity in large human studies [5,6], and higher consumption of the cysteine precursor methionine is associated with increased BMI and diabetes prevalence [7]. Phenotypes of genetic syndromes affecting cysteine metabolism (discussed in Ref. [6]) suggest that the relation of tCys with obesity reflects a causal role for cysteine in regulating body weight. In >5000 men and women, tCys was associated with waist–hip ratio, suggesting a relation between cysteine and increased visceral fat [5]. The mechanism underlying the association of tCys with human adiposity is unknown. *In vitro* l-cysteine inhibits rat adipocyte lipolysis in concentrations of 0.1–0.5 mmol/L, over 10 times the circulating concentrations of free cysteine in humans [8]. Direct *in vivo* evidence implicating cysteine in promoting obesity or metabolic risk is lacking.

Indirect evidence on involvement of cysteine in body fat regulation and glucose homeostasis comes from effects of dietary methionine restriction, which decreases plasma tCys by nearly one half [9,10]. Two different low-methionine dietary rodent models feature decreased weight gain and/or fat mass coupled with increased metabolic rate, with decreased plasma glucose and insulin concentrations [11,12]. The hypermetabolic phenotype in both models is linked to profound suppression of hepatic stearoyl CoA desaturase-1 (SCD-1), a δ-9 fatty acid desaturase regulated by sterol regulatory element binding protein-1 (SREBP-1) and which has emerged as a key regulator of lipid and energy metabolism [13]. Genetic SCD-1 deficiency in mice is linked to resistance to obesity [14] and improved insulin sensitivity [14], partly mediated *via* suppression of the negative regulator of insulin signaling, protein tyrosine phosphatase-1B (PTP-1B) [15].

In contrast to low-methionine diets, cysteine and cystine supplementation in rodent models increases weight gain despite reduced or unchanged food intake [16–18]. This pattern is consistent with early observations that sulfur amino acid content of diets correlates with food conversion efficiency (g weight gain/g food consumed) [19], and suggests an influence of cysteine on metabolic rate. The body composition, energy expenditure and metabolic features of cyst(e)ine-supplemented rodents are, however, unknown. We therefore investigated the metabolic phenotype associated with high vs. low cystine intakes in mice.

## 2 Methods

### 2.1 Animal husbandry and diets

Animal studies were conducted using guidelines issued by the Medical Research Council in “Responsibility in the Use of Animals for Medical Research” (July 1993). Mice were kept in accordance with UK Home Office welfare guidelines and project license restrictions under controlled light (12-h light and 12-h dark cycle; dark 7 p.m.–7 a.m.), temperature (21°C±2°C) and humidity (55%±10%) conditions. They had free access to water (10 ppm chlorine) and a commercial diet [SDS Rat and Mouse No.3 Breeding diet (RM3)] containing 3.36 g% fat, 22.45 g% protein and 71.21 g% carbohydrate.

Male C3H/HeH mice were maintained on RM3 diet from weaning till maturity. At 10 weeks of age, mice were shifted to either a low-cystine (LC) or high-cystine (HC) diet consisting of 10% casein protein diet with (HC) or without (LC) addition of 0.8 g% l-cystine for an experimental duration of 12 weeks. The LC diet had a calculated l-cystine content of 0.07 g%, methionine content of 2.9 g% and more than adequate protein content for maintenance requirements of adult mice [20] (Table 1) (D10012M/ AIN-93M Modified Rodent Diet, Research Diets, New Brunswick, NJ, USA). A third group was continued on the Breeding RM3 diet (standard diet; SD) for monitoring of weight gain compared to the LC and HC groups.

Phenotyping tests were performed according to the European Phenotyping Resource for Standardised Screens from EUMORPHIA standardized protocols as described at http://empress.har.mrc.ac.uk. Unless otherwise stated, phenotyping tests are described and reported with reference to the number of weeks on the experimental diets.

### 2.2 Body composition

Body mass was measured each week from 10 weeks of age on scales calibrated to 0.01 g. Analysis of body composition was performed by dual-energy x-ray absorptiometry (DEXA) scanning using the Lunar PIXImus Mouse Densitometer at weeks 0, 4, 8 and 12 of the experiment (Wipro GE Healthcare, Madison, WI, USA). Before DEXA analysis, a general anesthetic (100 mg/kg of ketamine, Pfizer; 10 mg/kg of xylazine, CEVA) was administered *via* intraperitoneal injection. After analysis, mice were revived by subcutaneous injection of atipamezole (1 mg/kg; Antisedan, Pfizer) and left to recover in a 37°C incubator for 1 h. Terminal dissection and measurement of fat pad weights were also performed.

### 2.3 Food consumption, plasma, liver and urinary parameters

At week 10, mice were placed in metabolic Techniplast cages with free access to water and food. Food consumption was measured by weighing. Urine was collected after 24 h in metabolic cages, and urinary catecholamines were measured using a 3-CAT Epinephrine, Norepinephrine, Dopamine enzyme-linked immunosorbent assay (ELISA) (Demeditec, Kiel-Wellsee, Germany) and were standardized against creatinine. Plasma leptin, insulin, adiponectin and glucagon levels were measured using a mouse endocrine MILLIPLEX kit (MILLIPLEX MAP, Millipore, Billerica, MA, USA) and a Bio-Plex 200 system (Bio-Rad, Hemel Hempstead, UK). At week 12, mice were fasted for 6 h during the light phase and given a lethal dose of anesthetic (200 mg/kg of ketamine, Vetalar, Pfizer, Kent, UK; 10 mg/kg of xylazine, Sedaxylin, CEVA, Buckinghamshire, UK) *via* intraperitoneal injection, and blood was collected by cardiac puncture. Plasma concentrations of glucose, lipids and albumin, and urine creatinine were measured on an AU400 (Olympus UK) as described [22,23]. Liver samples were homogenized in 5% Triton-X100 in dH_2_O, heated to above 80°C, cooled and heated again to solubilize triglyceride into solution. Liver lysate triglyceride, glycerol and free fatty acids were analyzed as per plasma samples [22].

### 2.4 Plasma sulfur amino acid and glutamate measurements

Liquid chromatography tandem mass spectrometry (LC-MS/MS) was used to analyze total homocysteine (tHcy), methionine, cystathionine, tCys and total glutathione (tGSH) [24].

#### 2.4.1 Analysis of taurine and glutamic acid by LC-MS/MS

Plasma (25 μl) was mixed with 25 μl internal standard solution containing 50 μmol/L [1,2-^13^C_2_]taurine (Isotec, Sigma Aldrich, Miamisburg, OH, USA) and 30 μmol/L ^2^H_3_-DL-glutamic acid (Cambridge Isotope Laboratories Inc., MA, USA) in 30 mmol/L ammonium formate, pH 3.2. Afterward, 125 μL methanol was added for protein precipitation, followed by centrifugation at 11,000*g* for 10 min. The supernatant was transferred to new vials and stored at 2°C until analysis. For separation, a Discovery HS F5 column (150×4.6 mm internal diameter, 3.5 μm pore size and a 2-μm precolumn filter, Sigma-Aldrich, Bellefonte, USA) was used. A Gilson 215 Autosampler (Middleton, WI, USA) was used to inject the sample (20 μL) onto the column, which had been equilibrated with a mobile phase (Perkin Elmer Series 200 Micro Pumps, Norwalk, CT, USA) composed of methanol and 30 mmol/L ammonium formate, pH 3.5, 60%:40% (v:v) at a flow rate of 1 mL/min. Separation was achieved by isocratic elution after 1.88 min for taurine, and 2.4 min for glutamic acid. Detection of taurine and glutamic acid was by multiple reaction monitoring on an API 365 PE SCIEX LC-MS/MS (Toronto, Canada) with Enhanced Performance system EP10+ (Bolton, Ontario, Canada), operating in the positive ion mode. Data acquisition and analysis were performed by Analyst 1.4.1 (MDS SCIEX, Applied Biosystems, Concord, Ontario, Canada). Interassay coefficient of variation for taurine and glutamic acid was 3.5% and 10.5%, respectively.

### 2.5 Metabolic rate

Metabolic rate was measured at week 4 using indirect calorimetry (Oxymax; Columbus Instruments, OH, USA) to determine oxygen consumption (VO_2_), carbon dioxide production (VCO_2_) and respiratory exchange ratio (RER). The VO_2_ and VCO_2_ were normalized to body mass and to lean and fat tissue mass derived from DEXA analysis.

### 2.6 Intraperitoneal glucose tolerance test

Each mouse was fasted overnight (16 h) to establish baseline glucose level “T0” (time zero). Mice were weighed, and a blood sample was collected from the tail vein after administration of local anesthesia (EMLA cream) using lithium-heparin Microvette tubes (Sarstedt, Nümbrecht, Germany). Each mouse then received an intraperitoneal injection of 2 g glucose/kg body weight (20% glucose in 0.9% NaCl). Subsequent blood samples were taken (at 60 and 120 min at week 7 or at 10, 20 and 20 min at week 11) after glucose injection. Plasma glucose was measured using an Analox Glucose Analyser GM9. Plasma insulin was measured using a Mercodia ultrasensitive mouse ELISA (Mercodia, Sweden). Area under the curve analysis was performed using GraphPad Prism version 5.02 for Windows.

### 2.7 Gene expression profiling

Total RNA was prepared from liver, gastrocnemius skeletal muscle and subcutaneous white adipose tissue (WAT) of LC (*n*=10) and HC (*n*=10) mice after 12 weeks on the diets following a 6-h light-phase fast.

Liver RNA was prepared using an RNeasy Plus Mini Kit (Qiagen, USA). Skeletal muscle RNA was prepared using an RNeasy fibrous Mini Kit, and WAT was prepared using an RNeasy Lipid Tissue Mini Kit (Qiagen, USA). The RNA concentration was assessed using a NanoDrop ND-1000 spectrophotometer (Thermo-Fischer Scientific). The ratio of absorbance at 260 and 280 nm was used to assess the purity of RNA. The extracted RNA was stored at −80°C. cDNA was prepared using Superscript III reverse transcriptase (Invitrogen). Quantitative polymerase chain reaction (PCR) was performed using TaqMan Gene Expression Assay reagents and TaqMan FAM dye-labeled probes (Applied Biosystems Inc., USA) using an ABIPRISM 7700 Fast Real-Time PCR System (Applied Biosystems Inc., USA). Data were normalized to expression of the endogenous housekeeping gene *glyceraldehyde 3-phosphate dehydrogenase* (*GAPDH*) and analyzed by the comparative ΔΔCT method to determine the difference between LC and HC groups.

### 2.8 Statistical methods

Data are expressed as mean±S.E.M. Comparison between groups was performed with a Student's *t* test for independent samples using Microsoft Excel 2003 for Windows. All tests were two-tailed, and *P*<.05 was considered statistically significant.

## 3 Results

### 3.1 Weight gain

**Fig. 1:**
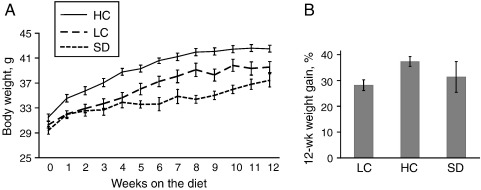
Body weight varies with dietary cystine. (A) Body weight of male mice on an LC diet (*n*=17 weeks 0–4, *n*=12 weeks 5–12), HC diet (*n*=17 weeks 0–4, *n*=12 weeks 5–12) and SD (*n*=10 weeks 0–4, *n*=5 weeks 5–12). Body weight in HC mice is significantly higher than that in LC mice (*P*<.05) at all time points except baseline and was significantly higher than that in SD mice at baseline (*P*=.019) and at all other time points (*P*<.05). Body weight in LC is significantly higher than that in SD only at weeks 8 and 10 (*P*<.05). (B) Percent weight gain after 12 weeks in the groups specified above. Percent body weight gain after 12 weeks is 33% higher in HC than in LC mice (*P*=.004). Percent weight gain in SD mice did not differ from that in LC or HC group (*P*≥.23). Data are expressed as mean±S.E.M.

The HC group gained consistently more weight than LC over the 12 weeks of the study (Fig. 1). At week 12, the percent weight change from baseline in the HC group was 33% higher than in the LC group (*P*=.004).

Body weight in LC and HC groups over the 12 weeks matched or exceeded the standard diet group (Fig. 1), confirming that the protein intake in LC and HC groups was adequate for maintenance requirements in these mature mice as recommended by Tobin et al. [20]. Subsequent analysis was restricted to comparing the LC and HC groups.

### 3.2 Body composition and plasma and hepatic lipids

**Fig. 2:**
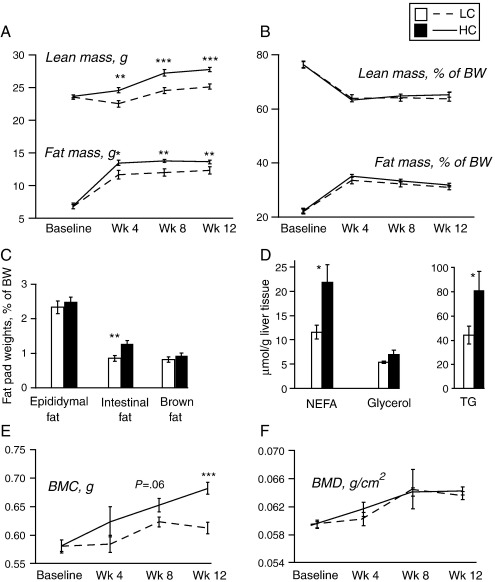
Body composition varies with dietary cystine. Body composition and related parameters in adult male mice on LC (*n*=12) and HC (*n*=12) diets. (A, B) Total fat mass and lean mass in grams and as a percent of body weight (BW) by DEXA scanning at baseline and weeks 4, 8 and 12 of the experiment. (C) Fat pad weights as a percent of total body weight by dissection and weighing at week 12. (D) Concentration of hepatic triglycerides (TG), nonesterified fatty acids (NEFA) and glycerol per gram of tissue at week 12. (E, F) Bone mineral content (BMC) and bone mineral density (BMD) by DEXA scanning at baseline and weeks 4, 8 and 12 of the experiment. Data are expressed as mean±S.E.M. ⁎*P*<.05, ⁎⁎*P*<.01, ⁎⁎⁎*P*<.001.

Total fat mass and lean mass measured by DEXA were similar in HC and LC groups at baseline, but were significantly higher in HC at weeks 4, 8 and 12 (Fig. 2A). Total fat mass as a percent of body weight remained similar in both groups, whereas peri-intestinal fat mass dissected at the termination of the study (week 12) was ∼30% higher in HC whether normalized to total fat mass or body weight (*P*=.003) (Fig. 2B, C). There was no difference in the weights of epididymal or brown fat pads. The HC mice also had elevated hepatic triglycerides and free fatty acids at week 12 (Fig. 2D).

At week 12, HC mice had higher plasma low-density lipoprotein cholesterol (LDL-C) and triglycerides than LC mice. Plasma glycerol was significantly reduced in HC mice, suggesting decreased lipolysis, but there was no difference in free fatty acid concentrations (Table 2). Similarly, there was no difference in plasma total cholesterol, high-density lipoprotein cholesterol (HDL-C) or leptin between the groups (Table 2).

A consistent increase in bone mineral content (BMC) measured by DEXA was seen in HC relative to LC, which reached statistical significance at week 12. There was no difference in bone mineral density (BMD) at the four time points examined (Fig. 2E, F).

### 3.3 Metabolic rate

**Fig. 3:**
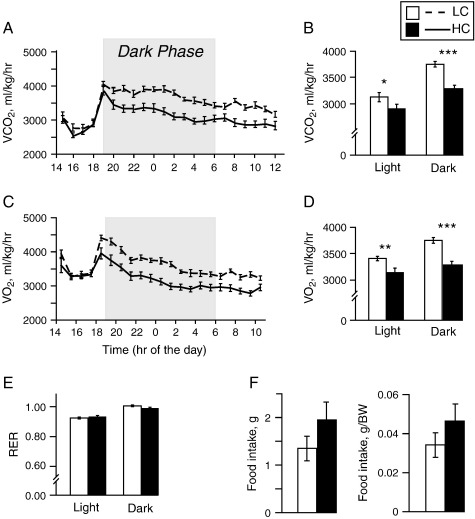
High dietary cystine reduces metabolic rate. (A, C) The VCO_2_ (A) and VO_2_ (C) during the light and dark phases for adult male mice on LC (*n*=17) and HC diet (*n*=17) normalized to body weight. (B, D) Total VCO_2_ (B) and VO_2_ (D) during the 22-h period. (E) The RER calculated from data given in A and C. (F) Food consumption during 24 h in grams (*P*=.20) and normalized to body weight (*P*=.27). Data are expressed as mean±S.E.M. ⁎*P*<.05, ⁎⁎*P*<.01, ⁎⁎⁎*P*<.001.

The VO_2_ and VCO_2_ measured at week 4 were markedly lower in the HC compared to the LC group during both the light and dark phases (*P*≤.001 for sum of both phases), whether normalized to body weight (Fig. 3A-D) or to lean mass or fat mass measured by DEXA at week 4 (data not shown). The RER, however, remained similar in both groups, suggesting that dietary cystine suppresses metabolic rate but does not influence the type of substrate (fat or carbohydrate) utilized for energy (Fig. 3E).

### 3.4 Food/water intake and urine parameters

Measurement of 24-h cumulative food intake in metabolic cages at week 10 showed that, although no significant difference was observed, HC showed a trend towards higher food consumption, even when adjusted for their higher body weight (Fig. 3F). Other metabolic cage parameters, namely, water consumption and urine output, were similar in both groups (data not shown).

Urine collected at week 10 was used to measure glucose, adrenaline and noradrenaline. There were no glucosuria in either group and no difference in urinary catecholamines between the LC and HC groups (data not shown).

### 3.5 Glucose homeostasis

**Fig. 4:**
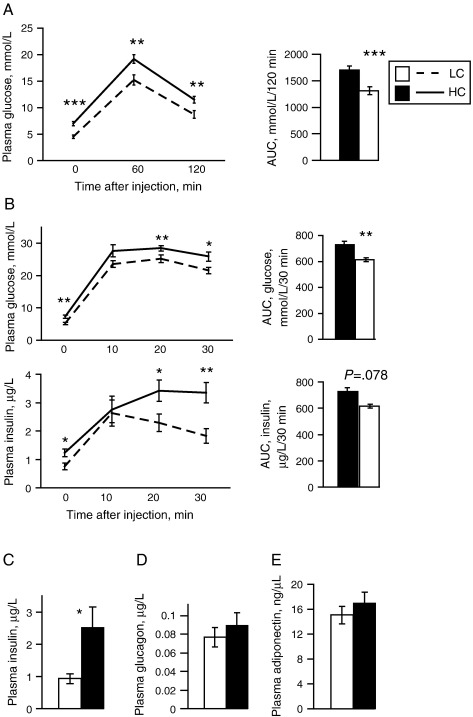
Effects of dietary cystine on glucose homeostasis. (A) A 120-min IPGTT following overnight fasting in adult male mice on LC (*n*=12) and HC (*n*=12) diets after 7 weeks on the diets. (B) A 30-min IPGTT following overnight fasting for plasma glucose and insulin in adult male mice on LC (*n*=12) and HC (*n*=12) diets after 11 weeks on the diets. (C, D, E) Plasma insulin (C), glucagon (D) and adiponectin (E) after 12 weeks on the diets. Data are expressed as mean±S.E.M. ⁎*P*<.05, ⁎⁎*P*<.01, ⁎⁎⁎*P*<.001.

Consistent with the increased visceral fat %, the HC group exhibited impaired glucose tolerance compared to LC. A 120-min intraperitoneal glucose tolerance test (IPGTT) performed at week 7 showed elevated 16-h fasting glucose in the HC group (7.0 vs. 4.5 mmol/L, *P*<.001), which remained significantly higher 60 and 120 min postinjection (Fig. 4A). A 30-min IPGTT at week 11 similarly showed reduced glucose tolerance in HC mice as evidenced by raised plasma insulin and glucose at baseline and at 20 and 30 min postinjection (Fig. 4B), suggesting the presence of insulin resistance.

The HC mice showed higher fasting plasma insulin than the LC mice at weeks 7, 11 and 12. There was no difference in plasma glucagon or adiponectin (measured at week 12) between the groups (Fig. 4C, D, E).

### 3.6 Plasma sulfur amino acids

Plasma methionine, tHcy, cystathionine, tCys and tGSH were measured at baseline and after 1, 2, 3, 4, 7 and 12 weeks on the diets, while taurine and glutamate were assayed at baseline and at weeks 1, 2, 3 and 12.

**Fig. 5:**
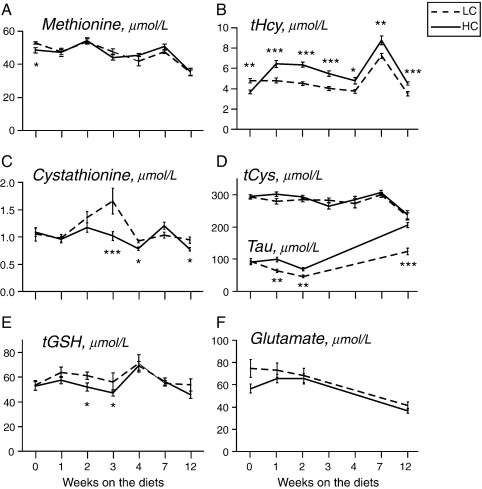
Effect of dietary cystine on plasma sulfur amino acids and glutamate. Plasma methionine, tHcy, tCys, taurine (Tau), tGSH and glutamate in adult male mice maintained on LC and HC diets for the durations shown. All measurements were done after a 6-h light phase fast except at week 7 (overnight 16-h fast). Number of mice per group: week 0, *n*=4; week 1, *n*=17; week 2, *n*=17; week 3: *n*=12; week 4, *n*=5; week 7, *n*=12; week 12, *n*=12. Data are expressed as mean±S.E.M. ⁎*P*<.05, ⁎⁎*P*<.01, ⁎⁎⁎*P*<.001.

There was no difference in plasma tCys between the HC and LC groups throughout the study, which was unexpected given the increase in dietary cystine in the HC group and similar food intake. However, plasma levels of taurine, a downstream product of cysteine, were consistently higher in the HC group at all time points examined apart from baseline, being ∼40% higher after 12 weeks on the diet (Fig. 5D).

The HC diet also produced elevation of tHcy (Fig. 5B), which persisted throughout the study, together with decrease in cystathionine (Fig. 5C). There was no consistent difference in tGSH, methionine or glutamate between the groups.

### 3.7 Gene expression profile

We compared the expression pattern of genes implicated in glucose, lipid and energy metabolism in liver, subcutaneous WAT and muscle in HC vs. LC mice, with focus on genes that have shown changes in response to methionine restriction [25].

**Fig. 6:**
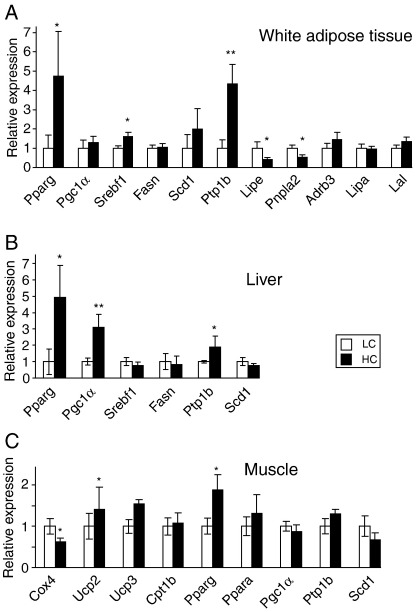
Effects of dietary cystine on gene expression. Gene expression levels, relative to GAPDH, measured by q-RT-PCR of metabolic genes in white adipose tissue (A), liver (B) and skeletal muscle (C) in adult male mice on LC (*n*=10) and HC (*n*=10) diets. Data are expressed as mean±S.E.M. ⁎*P*<.05, ⁎⁎*P*<.01.

In WAT, both the basal lipolytic enzyme adipose triglyceride lipase (ATGL, *Pnpla2*) and catecholamine-stimulated hormone sensitive lipase (HSL, *Lipe*) were suppressed in HC compared to LC, with no effect on the β3 adrenoceptor (*Adrb*3) (Fig. 6A). There was no effect of dietary cystine on lysosomal acid lipase (LAL, *Lipa*), which mainly mediates hydrolysis of cholesterol esters, or lipoprotein lipase (LL, *Lpl*), which hydrolyses triglycerides in circulating lipoproteins for uptake by adipose tissue (Fig. 6A).

In WAT, high dietary cystine also induced the transcription factors peroxisome proliferator-activated receptor-γ (PPAR-γ) (*Pparg*) and SREBP-1 (*Srebf1*), but had no significant effect on expression of the SREBP-target genes SCD-1 or fatty acid synthase (FAS, *Fasn*). Consistent with the reduced glucose tolerance in HC mice, the negative regulator of insulin signaling, PTP-1B (*Ptpn1*), was up-regulated by ∼fourfold in these mice compared to LC group (Fig. 6A).

Similar to WAT changes, hepatic PPAR-γ and PTP-1B gene expression was also up-regulated in HC mice, with no difference in SCD-1 or SREBP-1 expression between HC and LC mice (Fig. 6B). However, in contrast to WAT, hepatic PPAR-γ co-activator α (PGC-1α, *Ppargc1a*) was induced by cystine feeding.

To further explore the mechanism of the hypometabolic phenotype of HC mice, we investigated the expression of genes involved in oxidative phosphorylation and energy metabolism in skeletal muscle. The nuclear encoded subunit of cytochrome *c* oxidase, cyclooxygenase 4 (COX 4, *COX4*), was significantly suppressed (*P*=.037) in HC mice by ∼40%, while uncoupling protein-3 (UCP-3) was induced, with no difference in UCP-2 (Fig. 6C). In contrast to WAT and liver changes, there was no significant difference in muscle PTP-1B expression. There was also no change in expression of SCD-1 or of carnitine palmitoyl acyltransferase 1b (CPT-1b), the rate-limiting enzyme in long-chain fatty acid oxidation in muscle mitochondria (Fig. 6C).

## 4 Discussion

We compared the metabolic and anthropometric phenotypes of mice with low and high cystine intakes. High dietary cystine decreased metabolic rate, lowered insulin sensitivity and increased visceral fat deposition, in conjunction with changes in expression of several genes involved in lipid and glucose metabolism. Cystine supplementation enhanced total fat mass, lean mass and BMC, with no effect on BMD. Fasting plasma tCys and tGSH did not increase, but plasma taurine was higher in the cystine-supplemented group throughout the study.

### 4.1 Dietary cystine and body composition

Cystine feeding enhanced fat mass and lean mass growth, with no net change in body fat % However, body fat distribution was shifted towards visceral fat accumulation. The visceral fat proportion of total body fat was increased, with increased hepatic triglycerides. Consistent with these findings, rats with decreased plasma tCys secondary to methionine restriction have a 30% reduction in visceral fat % [9].

The mechanism of increased fat mass needs to be further investigated, but suppression of expression of both HSL and ATGL in HC mice, if translated into decreased levels of these enzymes, could suggest that high cystine intake may inhibit adipose tissue lipolysis, as seen *in vitro* in response to cysteine [8]. Decreased expression of HSL and ATGL is a feature of human obesity and insulin resistance [26,27], although it is debated whether suppression of lipolysis is the cause or a consequence of the obese state [28]. Another factor underlying increased adiposity in the HC group is decreased energy expenditure.

### 4.2 Dietary cystine and metabolic rate

Dietary cystine substantially suppressed metabolic rate. In line with this, dietary cyst(e)ine increases weight gain in the face of reduced [18] or unchanged [17] food intake, suggesting that cysteine affects the energy expenditure arm of the energy balance equation. Neither urinary catecholamines nor WAT B3-adrenoceptor expression was altered in HC mice, suggesting that the reduced metabolic rate is mediated by local tissue changes rather than altered sympathetic signal. There was also no appreciable change in physical activity, but that was not formally measured. However, as the metabolic rate differences were noted in both the dark (active) and light phases, physical activity is unlikely to be a major contributor to these differences.

Hepatic SCD-1 suppression is linked to hypermetabolic phenotypes associated with dietary restriction of the cysteine precursor methionine [12,25], and responds to cysteine supplementation of methionine-restricted rats [29]. We therefore hypothesized that the effect of cystine on metabolic rate in our model could be mediated by SCD-1. However, we did not note any changes in SCD-1 expression in liver, muscle or adipose. This does not exclude that SCD-1 protein levels may be altered, as seen in methionine restriction [25]. There was similarly no consistent effect of HC on expression of uncoupling proteins. The HC mice, however, featured enhanced PTP-1B expression by fourfolds in WAT and twofolds in the liver. Protein tyrosine phosphatase-1B, a phosphatase physiologically involved in terminating the insulin signaling cascade [30], is overexpressed in obesity and insulin resistance [31]. Notably, PTP-1B is also involved in regulation of energy expenditure, and PTP-1B-deficient mice feature increased energy expenditure and decreased adiposity independent of uncoupling protein expression [32]. This suggests that PTP-1B induction may be implicated in the decreased energy expenditure and increased adiposity of HC mice, as well as in their lowered insulin sensitivity, as discussed below. Finally, products of cysteine, including H_2_S and taurine, have both been found in animal models to decrease metabolic rate [33,34]. Thus, an effect of these products on metabolic rate in HC mice cannot be excluded, given their elevated plasma taurine.

### 4.3 Dietary cystine and glucose/lipid homeostasis

Dietary cystine adversely affected fasting plasma glucose and insulin, and 30- and 120-min intraperitoneal glucose tolerance. We postulate that this is related to the enhanced expression of hepatic and WAT PTP-1B, a negative regulator of insulin signaling [30]. Hepatic PTP-1B overexpression produces not only insulin resistance but also hypertriglyceridemia resulting from induction of hepatic SREBP-1 [35,36]. This parallels the SREBP-1 overexpression and increased plasma triglycerides observed in the HC group. Hepatic PTP-1B overexpression is also implicated in assembly and secretion of apolipoprotein B (ApoB)-containing lipoproteins [37], which may explain the cystine-induced elevation of the ApoB-related LDL-C in the present study. Increased plasma ApoB in response to a high-cystine diet has been previously reported [38], as well as increased hepatic and plasma total cholesterol [39]. In humans, plasma tCys positively correlates with plasma total cholesterol [40] and fasting serum LDL-C and ApoB [1]. Thus, combined evidence from human and rodent studies suggests that increased cystine availability promotes an insulin-resistant and dyslipidemic state.

In apparent contradiction to our findings, l-cysteine supplementation improved glucose tolerance in a rat model of type 2 diabetes [41], and a cysteine-rich protein supplementation alleviated sucrose-induced insulin resistance [42]. However, these effects were observed in diseased models rather than in normal mice as in our study. Few studies have examined the effect of cysteine administration on glucose homeostasis in conditions of normal glucose tolerance. In one study, glucose tolerance deteriorated in rats following a single intravenous injection of cysteine [43]. In the present study, it is likely that the increased visceral fat mass contributed to the insulin resistance phenotype. Increased intrahepatic fat, as observed in HC mice, was shown to be an even better predictor of metabolic deterioration than visceral fat [44].

### 4.4 Dietary cystine and plasma sulfur amino acids

In humans, plasma tCys is a strong predictor of BMI and fat mass [5,6,40], but determinants of plasma tCys variability are yet to be investigated. Epidemiologic predictors, rather than causal biologic determinants of tCys, have been reported [40] and include blood pressure, plasma cholesterol and BMI. Cysteine and its precursor, methionine, are ingested in diet, but there is some evidence to suggest that dietary cyste(i)ne intake may not be an important determinant of tCys. In a recent study in 812 healthy women, plasma tCys was unrelated to dietary intakes of either cystine or methionine [45]. A similar observation was made in cats fed different levels of cysteine [46]. One factor explaining the lack of association of plasma tCys with cystine intake may be the central role of the liver in disposing of excess dietary cysteine through conversion to taurine [47]. Indeed, in the present study, the HC group did not have higher plasma tCys but had higher plasma taurine throughout the study. Similarly, increasing dietary cystine intake in growing rats [48] raised plasma taurine concentrations but did not raise plasma cystine. On the other hand, Kawakami et al. [16] reported modest increases in plasma tCys in response to supplementation of cysteine in its reduced form. One reason for the discrepancy with our findings besides the different form of cysteine supplemented is that Kawakami et al. used nonfasting samples, which may have enabled the detection of a transient postprandial rise in tCys.

Dietary cystine supplementation had a minimal effect on plasma concentrations of other sulfur amino acids, with the notable exception of the consistently higher plasma tHcy in the HC group. We speculate that this could be explained by inhibition of transsulfuration, as evidenced also by the decreased plasma concentrations of the first transsulfuration product, cystathionine. Finkelstein et al. [49] demonstrated suppression of transsulfuration and of cystathionine formation when supplementing rats on a methionine-free diet with cystine, in a recognized “methionine-sparing effect of dietary cystine.”

## 5 Summary

In summary, a high-cystine diet in adult mice increased growth and visceral fat, suppressed metabolic rate and decreased glucose tolerance, suggesting a central role of cysteine in metabolic regulation. Given the established link of plasma tCys with obesity [5,6] and metabolic syndrome [3] in epidemiologic studies, the role of increased cysteine availability in pathogenesis of human obesity and insulin resistance should be further investigated.

The following are the supplementary materials related to this article.

Supplementary Figure 1 Cysteine metabolic pathways. The body cysteine pool is the product of protein turnover, dietary intake and synthesis from methionine via two intermediates: homocysteine and cystathionine. Cysteine is also used in synthesis of coenzyme A by condensation with pantothenic acid and synthesis of glutathione via condensation with glycine and glutamate. Excess cysteine is converted to taurine mainly in the liver. Compounds shown in bold have been measured in plasma in the present study. Dotted lines show pathways with omitted intermediates for purposes of clarity.

## Figures and Tables

**Table 1:** Composition of the experimental diets

Ingredient, g%	LC	HC
Casein	10	10
*Contains: l-cystine*	*0.07*	*0.07*
*Contains: methionine*	*0.29*	*0.29*
l-Cystine (supplemented)	0	0.8
Cornstarch	53.7	52.9
Maltodextrin 10	12.5	12.5
Sucrose	10	10
Soybean oil	4	4
*Total protein*	10	10.8
*Total carbohydrate*	77	76.2
*Total fat*	4	4

Other constituents common to both diets include cellulose (5 g%), mineral mix (3.5 g%), vitamin mix (0.1 g%) and choline bitartrate (0.25 g%). Vitamin and mineral mixes were formulated according to American Institute of Nutrition guidelines [21].

**Table 2:** Effect of dietary cystine on plasma metabolic parameters

	LC (*n*=12)	HC (*n*=11)	*P* value
TG, mmol/L	1.14±0.09	1.56±0.14	***.014***
FFA, mmol/L	1.07±0.06	0.98±0.07	*.34*
Glycerol, μmol/L	613±34	492±30	***.014***
Total-C, mmol/L	5.42±0.23	6.13±0.32	*.084*
HDL-C, mmol/L	3.60±0.32	3.41±0.21	*.64*
LDL-C, mmol/L	0.56±0.03	0.88±0.14	***.026***
Glucose, mmol/L	16±1	16±2	*.97*
Albumin, g/L	27.9±0.5	25.9±0.8	*.22*
Leptin, ng/mL	13.2±2.8	17.0±2.8	*.35*

In 6-h fasting adult male mice after 12 weeks on the diets. Data are given as mean±S.E.M. TG, triglycerides; FFA, free fatty acids; Total-C, total cholesterol; HDL-C, HDL cholesterol; LDL-C, LDL cholesterol.
