# Supplementary material for: Dietary cystine level affects metabolic rate and glycaemic control in adult mice
Source: J Nutr Biochem. 2012 Apr;23(4-2):332–40. doi: 10.1016/j.jnutbio.2010.12.009 (PMC3315011; doi:10.1016/j.jnutbio.2010.12.009)
Supplement: Supplementary Figure 1 — Cysteine metabolic pathways. The body cysteine pool is the product of protein turnover, dietary intake and synthesis from methionine via two intermediates: homocysteine and cystathionine. Cysteine is also used in synthesis of coenzyme A by condensation with pantothenic acid and synthesis of glutathione via condensation with glycine and glutamate. Excess cysteine is converted to taurine mainly in the liver. Compounds shown in bold have been measured in plasma in the present study. Dotted lines show pathways with omitted intermediates for purposes of clarity. [file mmc1.pdf]

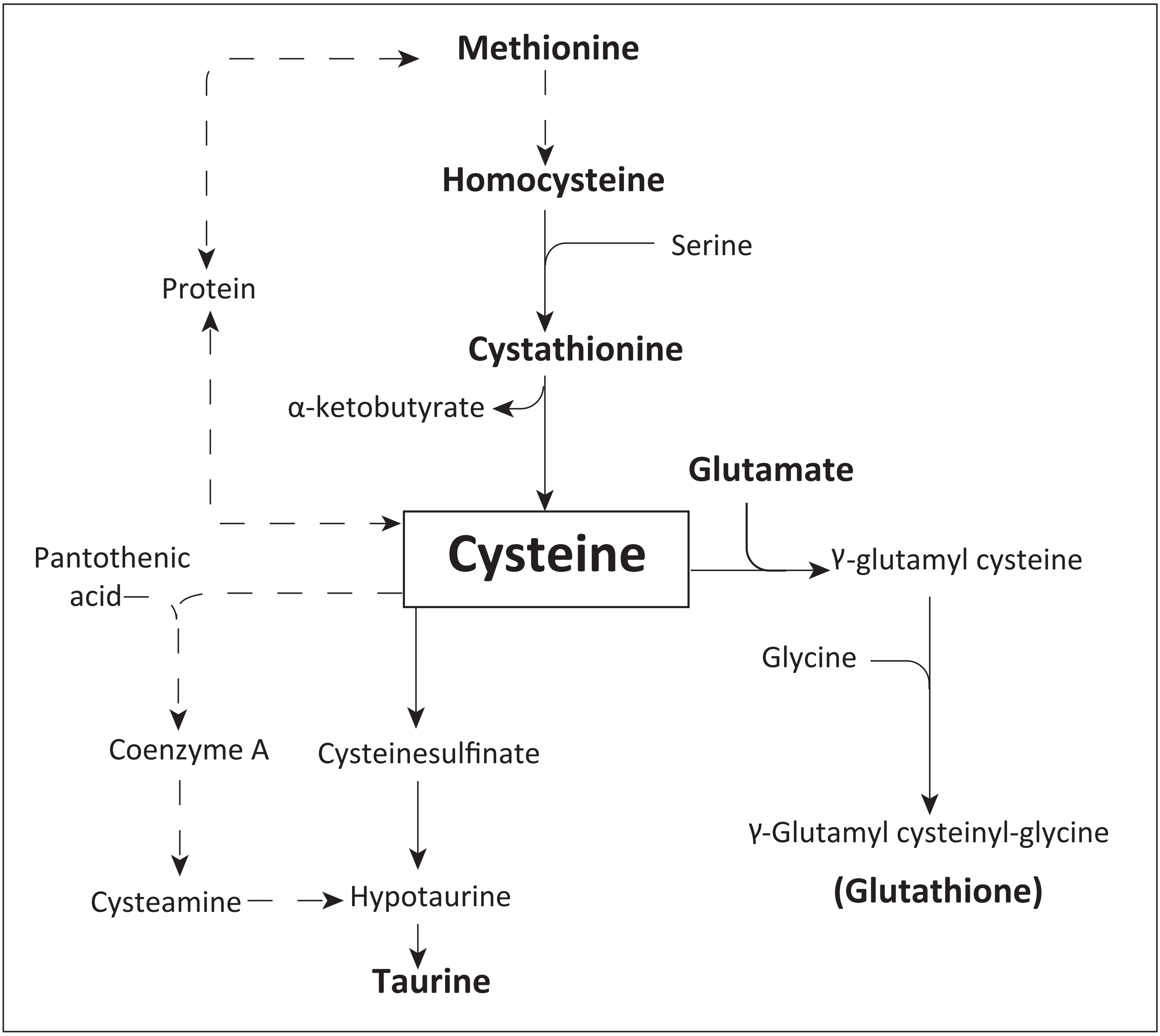

Supplementary Figure 1

Cysteine metabolic pathways. The body cysteine pool is the product of protein turnover, dietary intake and synthesis from methionine, via 2 intermediates, homocysteine and cystathionine. Cysteine is also used in synthesis of coenzyme A by condensation with pantothenic acid, and synthesis of glutathione, via condensation with glycine and glutamate. Excess cysteine is converted to taurine mainly in the liver. Compounds shown in bold have been measured in plasma in the present study. Dotted lines show pathways with omitted intermediates for purposes of clarity.
